# Premature timing of progesterone luteal phase support initiation did not negatively impact live birth rates in modified natural frozen thawed embryo transfer cycles

**DOI:** 10.1016/j.heliyon.2023.e13218

**Published:** 2023-01-24

**Authors:** Wen-Jing Jiang, Zhen-Gao Sun, Jing-Yan Song

**Affiliations:** a The First Clinical College, Shandong University of Traditional Chinese Medicine, Jinan, China; b Reproductive Center of Integrated Medicine, The Affiliated Hospital of Shandong University of Traditional Chinese Medicine, Jinan, China

**Keywords:** Modified natural cycle/frozen-thawed embryo transfer/human chorionic gonadotropin /luteinizing hormone/in vitro fertilization and embryo transfer

## Abstract

**Study question:**

In a modified natural cycle frozen-thawed embryo transfer (mNC-FET), does the premature timing of progesterone luteal phase support (LPS) initiation 24 h following human chorionic gonadotropin (hCG) trigger impact live birth?

**Summary answer:**

Premature LPS initiation did not negatively affect the live birth rate (LBR) in mNC-FET cycles compared with conventional LPS initiation 48 h after hCG triggering.

**What is known already:**

During natural cycle FET, human chorionic gonadotropin is routinely used to mimic endogenous luteinizing hormone (LH) surge to induce ovulation, which allows more flexibility in embryo transfer scheduling, thus relieving the burden of multiple visits by patients and laboratory workloads, which is also known as mNC-FET. Moreover, recent data demonstrates that ovulatory women undergoing natural cycle FETs have a lower risk of maternal and fetal complications due to the essential role of the corpus luteum in implantation, placentation and pregnancy maintenance. While several studies have confirmed the positive effects of LPS in mNC-FETs, the timing of progesterone LPS initiation is still unclear, as compared with fresh cycles where robust research has been conducted. To the best of our knowledge, no clinical studies comparing different beginning days in mNC-FET cycles have been published.

**Study design, size, duration:**

This retrospective cohort study involved 756 mNC-FET cycles performed at a university-affiliated reproductive center between January 2019 and August 2021. The primary outcome measured was the LBR.

**Participants/materials, setting, methods:**

Ovulatory women ≤42 years of age who were referred for their autologous mNC-FET cycles were included in the study. According to the timing of progesterone LPS initiation following the hCG trigger, patients were assigned into two categories: premature LPS group (progesterone initiation 24 h after hCG trigger, n = 182) versus conventional LPS group (progesterone initiation 48 h after hCG trigger, n = 574). Multivariate logistic regression analysis was used to control for confounding variables.

**Main results and the role of chance:**

There were no differences in background characteristics between the two study groups, except for the proportion of assisted hatching (53.8% in premature LPS group versus 42.3% in conventional LPS group, p = 0.007). In the premature LPS group, 56 of 182 patients (30.8%) had a live birth, compared to 179 of 574 patients (31.2%) in the conventional LPS group, with no significant difference observed between groups (adjusted odds ratio [aOR] 0.98, 95% confidence interval [CI] 0.67–1.43, p = 0.913). In addition, there was no significant difference between the two groups in other secondary outcomes. A sensitivity analysis for LBR according to the serum LH and progesterone levels on hCG trigger day also confirmed the aforementioned findings.

**Limitations, reasons for caution:**

In this study, retrospective analysis was conducted in a single center and was therefore prone to bias. Additionally, we did not anticipate monitoring the patient's follicle rupture and ovulation after hCG triggering. Future prospective clinical trials remain necessary to confirm our results.

**Wider implications of the findings:**

While exogenous progesterone LPS was added 24 h after hCG triggering, embryo-endometrium synchrony would not be adversely affected so long as sufficient time was allowed for endometrial exposure to exogenous progesterone. Our data support promising clinical outcomes following this event. As a result of our findings, clinicians and patients will be able to make better informed decisions.

**Study funding/competing interest(s):**

No specific funding was available for this study. The authors have no personal conflicting interests to declare.

**Trial registration number:**

N/A.

## 1 Introduction

In the realm of assisted reproduction, embryo freezing and frozen-thawed embryo transfer (FET) have been extensively employed since the first embryo freezing, resuscitation, and transfer to achieve pregnancy in 1983. Frozen-thawed embryo transfer can successfully minimize the risk of ovarian hyperstimulation syndrome (OHSS), reduce the number of ovarian stimulation cycles, lower the danger of ectopic pregnancy, and maximize the cumulative pregnancy rate. Thus, the proportion of FETs in embryo transfer cycles has been increasing rapidly over the past few years [1]. Currently, natural cycle (NC-FET), hormone replacement treatment cycle (HRT-FET), and stimulation cycles are the most frequently employed endometrial preparation regimens in clinical practice. However, there is no consensus on the optimal endometrial preparation protocol [2]. Notably, NC-FET is indicated for patients who experience regular ovulation [3,4]. Although NC-FET does not require exogenous hormones and makes the process physiologically similar to the patients' natural states, it has the disadvantages of repetitive monitoring and high cycle cancellation rate [5]. Hence, in clinical practice, human chorionic gonadotropin (hCG) is routinely used to simulate endogenous LH surge to induce ovulation, which is also known as mNC-FET. Compared to NC-FET, mNC-FET minimized the need for ultrasound and serum hormone monitoring, shortened the monitoring period, made it easier for clinicians to schedule the timing of embryo transfer, and decreased cycle cancellation rates [6,7]. Furthermore, it improved patient acceptability and economic cost-effectiveness.

Although NC-FET or mNC-FET patients have regular monthly cycles and normal serum progestogen (P4) levels, some still experience luteal phase defects (LPD), which are aberrant endometrial responses to acceptable amounts of P4 exposure or inadequate P4 generation during the luteal phase, which lead to unsuccessful embryo implantation and early abortion [8]. According to recent studies, luteal phase support (LPS) enhances the success of mNC-FET pregnancies [[9], [10], [11]]. In terms of the optimal timing for administering P_4_ in mNC-FET, there is disagreement, and local clinic practices differ significantly throughout the globe. In clinical practice, most investigators believe that ovulation occurs 36–48 h after hCG injection, so current studies often start using P_4_ for LPS 48 h after hCG triggering [9,10,12,13], and some investigators administer it 36 h after hCG triggering [14,15]. However, it has been shown that hCG triggering leads to an increase in progesterone in the early luteal phase [16,17,18], which advances the implantation window and leads to asynchrony between the embryo and endometrium, which may lead to implantation failure [19,20]. Therefore, a limited number of researchers have decided that P_4_ administration should take place 24 h after hCG triggering in order to prevent the issue of decreased endometrial receptivity to embryos brought on by early elevations of P_4_ following hCG triggering [21]. Additionally, no comparative studies have been done on the most appropriate time to provide P_4_ LPS during mNC-FET cycles.

Therefore, the purpose of this study was to retrospectively assess the relationship between the timing of the administration of P4 LPS and reproductive outcomes in mNC-FET and to explore the optimal timing of P4 initiation for providing clinicians and patients with more options.

## 2 Materials and methods

### 2.1 Study Design

**Fig. 1:**
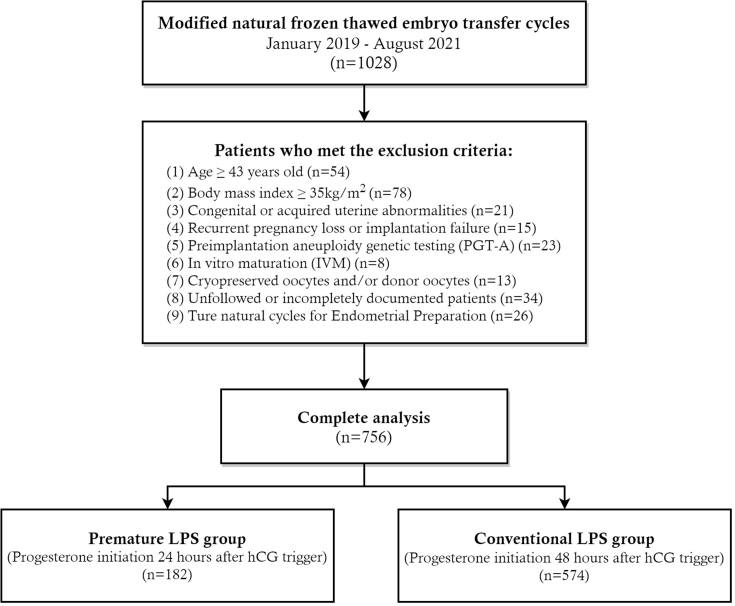
**Flowchart of the study cohort of premature LPS versus conventional LPS in modified natural frozen thawed embryo transfer cycles.** LPS, luteal phase support; hCG, human chorionic gonadotropin.

Between January 2019 and August 2021, 756 mNC-FET cycles performed at the investigator's reproductive center were included in this retrospective cohort study (see Fig. 1). The study was authorized by the local institutional review board (Reproductive Ethics Committee of the Affiliated Hospital of Shandong University of Traditional Chinese Medicine, SDTCM/E2208-01, dated August 15, 2022) and was carried out in a public tertiary care hospital.

### 2.2 Eligibility criteria

During this study, ovulatory women undergoing autologous mNC-FET cycles with an age younger than 42 years old were included. The following patients were not included in this investigation: (1) body mass index (BMI) ≥ 35 kg/m^2^; (2) congenital or acquired uterine abnormalities; (3) recurrent pregnancy loss and recurrent implantation failure; (4) preimplantation aneuploidy genetic testing (PGT-A); (5) in vitro maturation (IVM); (6) cryopreserved oocytes and/or donor oocytes; (7) unfollowed or incompletely documented data. Frozen-thawed embryo transfer with true natural and hormone-replacement cycles were also disregarded.

Included patients were divided into two groups of premature LPS group (progesterone initiation 24 h after hCG trigger, n = 182) and conventional LPS group (progesterone initiation 48 h after hCG trigger, n = 574). Premature LPS patients began receiving exogenous progesterone for luteal phase support (LPS) on the first day after hCG triggering, whereas conventional LPS patients begin 48 h after triggering.

### 2.3 Ovarian stimulation and laboratory procedures

Patients underwent ovarian stimulation with gonadotropin-releasing hormone agonist (GnRH-a), GnRH antagonist (GnRH-ant), or mini-stimulation protocol as previously described, and oocyte retrieval via ultrasound-guided transvaginal ovarian puncture is carried out 36–37 h after hCG (Ovitrelle®, 250 μg, Merck) or GnRH-a combined with hCG (double-trigger) [22,23]. Following egg retrieval, fertilization was carried out using intracytoplasmic sperm injection (ICSI) or in vitro fertilization (IVF) depending on the quality of the male sperm. Under ultrasound guidance, a fresh embryo transfer was carried out, and any remaining good-quality embryos or blastocysts were vitrified. Alternatively, if clinically indicated, a freeze-all strategy was employed. Good-quality D3 embryos had ≥6 blastomeres and ≤20% fragmentation. Good-quality D5 blastocysts should have embryo scores ≥ 3BB. A detailed description of the vitrification and thawing processes can be found elsewhere [24]. The American Society for Reproductive Medicine committees’ opinions on guidance on the number of embryos to transfer were followed [25].

### 2.4 Endometrial preparation protocol

Transvaginal ultrasound and serum hormone measurements (luteinizing hormone (LH), estradiol (E_2_) and P_4_) were performed on days 8 through 10 of the menstrual cycle, depending on the duration of the menstrual cycle, to track endometrial thickness and follicle size until ovulation triggering conditions were reached. A single intramuscular injection of 8000 IU hCG (Lizhu Pharmaceutical Trading Co., China) was used to trigger ovulation when the dominant follicle diameter was more than 17 mm, the endometrial thickness was more than 7 mm, P_4_ was less than 1.5 ng/ml, and E_2_ was more than 150 pg/ml. At approximately 9:00 a.m., the hCG injection was administered. Patients who had unanticipated spontaneous ovulation while being monitored, as well as those who had no prominent follicles by day 25 of the menstrual cycle, were eliminated.

### 2.5 Luteal phase support protocol

In the conventional LPS group, P_4_ LPS started 48 h after hCG triggering. Exogenous P_4_ was started 24 h after hCG triggering in the premature LPS group. When LPS would be initiated was determined by the attending physician's clinical judgment, which was primarily determined by the specific day the physician was scheduled to perform the embryo transfer procedure, as well as avoiding weekends so that laboratory workload could be reduced. Progesterone (P_4_) was given intramuscularly (20 mg, Zhejiang Xianju Pharmaceutical Co., Ltd.), vaginally (90 mg, 8% Crinone, Moxerano), orally (10 mg, dydrogesterone, Abbott Laboratories biologicals), or in combination. The manner of administration is determined by the clinical preferences of both physicians and patients. D3 embryos or blastocysts were resuscitated for transfer on Day 4 or Day 6 after P_4_ administration. A prominent physician in the department performed the embryo transfer operation. After FET, daily P_4_ medication treatment was continued until the pregnancy test came out negative. Luteal phase support was continued if clinical pregnancy was confirmed until the placenta entirely replaced luteal function at 8–10 weeks of gestation.

### 2.6 Assessment of pregnancy outcomes

The primary outcome was the live birth rate (LBR), which was defined as the delivery of a viable newborn at 28 weeks or more gestation. The key secondary outcomes included the positive pregnancy rate, clinical pregnancy rate, embryo implantation rate, pregnancy loss rate, ectopic pregnancy rate, and multiple pregnancy rate (including twins). Gestational diabetes, gestational hypertension, preterm delivery, singleton birth weight, and small or large for gestational age are among the maternal and fetal outcomes monitored for more than 24 weeks of gestation.

### 2.7 Statistical analysis

The Shapiro-Wilk test was used to determine the data's normality. If the quantitative variables were regularly distributed, independent t-tests and means and standard deviations (SDs) within the groups of interest were utilized for comparisons. Where data did not have a normal distribution, the Mann-Whitney *U* test was used to compare median and interquartile range (IQR) among groups of interest. Pearson Chi-square or Fisher exact tests were used to compare qualitative variables, which included numerator and denominator values and were provided as a number or percentage of instances. We used multivariable logistic regression analysis to identify factors that could be linked with the LBR, with the LBR as the dependent variable and timing of LPS initiation after the hCG trigger in the mNC-FET as the key independent variable. Female age at oocyte retrieval, BMI, FSH and LH on menstrual cycle days 2–3, cycle outcomes, number of precious ET cycles, LH and P_4_ level on LH surge day, luteal phase support, P_4_ level on ET day, assisted hatching, embryo transfer stage, number of embryos transferred, good-quality embryo transferring, physicians of embryo transfer (A, B, C, D), and endometrial thickness were among the potential predictors taken into consideration in the analysis. With a 95% confidence interval (CI), the odds ratio (OR) was used to represent the risk of LBR. In order to confirm the reliability of the findings, we also carried out additional sensitivity analyses on LBR in both groups, taking into account the potential influence of LH (<15, 15–24.9, 25–39.9, ≥40 mIU/ml) and P_4_ (<1.0, ≥1.0 ng/ml) levels on the LH surge day [26]. The SPSS software, version 26.0, was used for all data analyses (SPSS Inc., Chicago, USA). All statistical tests had a two-tailed alpha of 0.05, and a P value < 0.05 was regarded as statistically significant for the two groups.

## 3 Results

### 3.1 Demographic and baseline IVF characteristics

The demographic and baseline IVF characteristics of 756 mNC-FET patients were investigated (Table 1). Luteal phase support was administered to 182 patients 24 h after hCG triggering, and to 574 patients 48 h afterwards. There were no significant differences between the two groups regarding demographics, cycle features, ovarian stimulation data, embryo outcomes, or cycle outcomes (P > 0.05).

**Table 1:** Demographic and baseline IVF characteristics of the study groups.

	Premature LPS (n = 182)	Conventional LPS (n = 574)	P-value
**Demographic characteristics**			
Female age at oocyte retrieval (years), mean ± SD	32.3 ± 3.9	32.6 ± 4.1	0.365
<35 yrs.	121 (66.5)	364 (63.4)	0.452
≥35 yrs.	61 (33.5)	210 (36.4)	
BMI (kg/m^2^), mean ± SD	22.6 ± 2.9	22.9 ± 2.9	0.296
**Cycle characteristics**			
Cause of infertility			0.289
Tubal factor	94 (51.6)	263 (45.8)	
Male factor	33 (18.1)	110 (19.2)	
Mixed factor	30 (16.5)	77 (13.4)	
Ovulation disorder/PCOS	8 (4.4)	54 (9.4)	
Endometriosis	9 (4.9)	41 (7.1)	
DOR or AMA	4 (2.2)	14 (2.4)	
Unexplained	4 (2.2)	15 (2.6)	
Duration of infertility (years), median (IQR)	3 (1, 4)	3 (2, 4)	0.512
Nulliparous	73 (40.1)	248 (43.2)	0.462
Gravidity, median (IQR)	1 (0, 2)	1 (0, 2)	0.990
Parity, median (IQR)	0 (0, 1)	0 (0, 1)	0.760
Basic FSH (mIU/ml), mean ± SD	7.4 ± 4.9	7.5 ± 3.1	0.863
Basic LH (mIU/ml), mean ± SD	5.0 ± 2.7	5.0 ± 2.6	0.981
Basic E_2_ (pg/ml), mean ± SD	40.6 ± 17.8	40.8 ± 17.7	0.903
**Ovarian stimulation data**			
Type of GnRH analog			0.152
GnRH-a	91 (50.0)	291 (50.7)	
GnRH-ant	80 (44.0)	223 (38.9)	
None (mini-stimulation)	11 (6.0)	60 (10.5)	
No of days of COS, mean ± SD	10.9 ± 2.3	10.7 ± 2.4	0.185
Total Gn dose administered (IU), median (IQR)	2475 (2025, 3066)	2325 (1800, 3000)	0.115
LH on trigger day (mIU/ml), median (IQR)	1.5 (1.0, 2.6)	1.7 (1.0, 2.8)	0.624
E_2_ on trigger day (pg/ml), mean ± SD	3604 ± 1332	3575 ± 1450	0.811
P_4_ on trigger day (ng/ml), median (IQR)	1.1 (0.8, 1.9)	1.1 (0.8, 1.5)	0.074
Trigger drugs			0.319
hCG	161 (88.5)	491 (85.5)	
GnRH-a and hCG	21 (11.5)	83 (14.5)	
**Embryologic outcomes**			
No of oocytes retrieved, mean ± SD	14.1 ± 7.7	14.0 ± 7.8	0.877
Fertilization methods			0.710
IVF	141 (77.5)	437 (76.1)	
ICSI	41 (22.5)	137 (23.9)	
2 PN zygotes, mean ± SD	8.9 ± 5.8	8.4 ± 5.2	0.253
Embryos available for transfer, mean ± SD	4.7 ± 2.4	4.5 ± 2.5	0.489
Blastocysts available for transfer, median (IQR)	2 (1, 3.5)	3 (2, 5)	0.227
No of high-quality embryos, median (IQR)	1 (0, 2)	1 (0, 2)	0.124
**Cycle outcomes**			0.436
Fresh ETs	44 (24.2)	123 (21.4)	
Freeze-all cycles	138 (75.8)	451 (78.6)	

**Abbreviation:** LPS, luteal phase support; DOR, diminished ovarian reserve; AMA, advanced maternal age; IQR, interquartile range; BMI, body mass index; AMH, anti-müllerian hormone; FSH, follicle stimulating hormone; LH, luteinizing hormone; E_2_, estradiol; P_4_, progesterone; GnRH-a, gonadotropin releasing hormone agonist; GnRH-ant, GnRH antagonist; IVF, in-vitro fertilization; ICSI, intracytoplasmic sperm injection; 2 PN, double pronuclear fertilization; Gn, gonadotropin; IU, international units.

Data are presented as numbers (%) unless otherwise noted.

### 3.2 Characteristics of the mNC-FET cycles

In terms of baseline mNC-FET characteristics (Table 2), there was a greater number of assisted hatching procedures in the premature LPS group than in the conventional LPS group (53.8% vs. 42.3%, P = 0.007). Nevertheless, there were no significant differences in the number of previous ET cycles, LPS protocol, serum E_2_, LH, P_4_ levels on LH surge day, serum E_2_, P_4_ levels on ET day, number of transferred embryos, embryo developmental stage, good-quality embryo transferring, physicians performing ET procedures, and endometrial thickness between the two groups (P > 0.05).

**Table 2:** Baseline mNC-FET characteristics of the study groups.

	Premature LPS (n = 182)	Conventional LPS (n = 574)	P-value
Number of previous ET cycles			0.199
1	83 (45.6)	310 (54.0)	
2	59 (32.4)	167 (29.1)	
3	25 (13.7)	56 (9.8)	
≥4	15 (8.3)	41 (7.1)	
Luteal support protocol			0.512
Injected progesterone	62 (34.1)	213 (37.1)	
Vaginal progesterone	39 (21.4)	135 (23.5)	
Dydrogesterone	23 (12.6)	76 (13.2)	
Mixed	58 (31.9)	150 (26.1)	
LH level on LH surge day (IU/L), median (IQR)	16.3 (8.6, 29.2)	16.3 (10.5, 32.1)	0.134
E_2_ level on LH surge day (pg/ml), median (IQR)	177 (107, 255)	160 (97, 250)	0.135
P_4_ level on LH surge day (ng/ml), median (IQR)	1.2 (0.8, 1.6)	1.3 (1.0, 1.6)	0.158
E_2_ level on ET day (pg/ml), median (IQR)	124 (100, 169)	134 (98, 193)	0.213
P_4_ level on ET day (ng/ml), median (IQR)	17.4 (13.3, 23.5)	17.7 (13.0, 23.1)	0.995
Assisted hatching	98 (53.8)	243 (42.3)	**0.007**
Number of transferred embryos			0.922
Single	45 (24.7)	144 (25.1)	
Double	137 (75.3)	430 (74.9)	
Embryo developmental stage			0.929
D3	30 (16.5)	93 (16.2)	
D5/D6	152 (83.5)	481 (83.8)	
Good-quality embryo transferring	84 (46.2)	256 (44.6)	0.713
Physicians performing ET procedures			0.585
Physician A	42 (23.1)	161 (28.1)	
Physician B	43 (23.6)	132 (23.0)	
Physician C	45 (24.7)	136 (23.7)	
Physician D	52 (28.6)	145 (25.3)	
Endometrial thickness (mm), mean ± SD	10.5 ± 1.7	10.3 ± 1.6	0.186

**Abbreviation:** mNC, modified natural cycle; LPS, luteal phase support; FET, frozen embryo transfer; IQR, interquartile range; LH, luteinizing hormone; E_2_, estradiol; P_4_, progesterone; SET, single embryo transfer; DET, double embryo transfer.

Data are presented as numbers (%) unless otherwise noted.

Statistically significant values are indicated in bold.

### 3.3 Pregnancy and birth outcomes

The primary outcome of LBR in the premature LPS group was non-inferior to the conventional LPS group (30.8% vs. 31.2%, OR 0.99, 95% CI 0.77–1.27, P = 0.916), as shown in Table 3. Furthermore, no significant differences were seen in positive pregnancy rate (39.6% vs.38.2%, OR 1.06, 95% CI 0.75–1.49, P = 0.734), clinical pregnancy rate (36.8% vs.37.6%, OR 0.97, 95% CI 0.68–1.36, P = 0.843), clinical pregnancy loss (18.1% vs.16.0%, OR 1.13, 95% CI 0.63–2.01, P = 0.681), ectopic pregnancy rate (4.2% vs.2.3%, OR 1.83, 95% CI 0.45–7.45, P = 0.665), multiple pregnancy rate (including twins) (15.3% vs.15.5%, OR 0.98, 95% CI 0.53–1.84, P = 0.960), preterm delivery (13.4% vs.12.5%, OR 1.08, 95% CI 0.53–2.17, P = 0.841), small for gestational age (5.4% vs.6.1%, OR 0.87, 95% CI 0.25–3.02, P > 0.999), large-for-gestational-age infants (10.7% vs. 8.9%, OR 1.20, 95% CI 0.49–2.92, P = 0.691), gestational diabetes (4.5% vs. 6.4%, OR 0.65, 95% CI 0.19–2.16, P = 0.663), and gestational hypertension (7.5% vs. 8.8%, OR 0.85, 95% CI 0.33–2.19, P = 0.732) between premature LPS and conventional LPS groups. In terms of embryo implantation rate and singleton birth weight, the independent *t*-test findings revealed no significant difference between the two groups (P > 0.05).

**Table 3:** Pregnancy and birth outcomes between the two study groups.

	Premature LPS (n = 182)	Conventional LPS (n = 574)	Odds ratio (95% CI)	P-value
Positive pregnancy rate	72 (39.6)	219 (38.2)	1.06 (0.75 to 1.49)	0.734
Clinical pregnancy rate	67 (36.8)	216 (37.6)	0.97 (0.68 to 1.36)	0.843
Embryo implantation rate (%), mean ± SD	25.3 ± 36.3	25.0 ± 35.8	–	0.928
Pregnancy loss per positive pregnancy	13 (18.1)	35 (16.0)	1.13 (0.63 to 2.01)	0.681
Ectopic pregnancy per positive pregnancy	3 (4.2)	5 (2.3)	1.83 (0.45 to 7.45)	0.665
Live birth rate	56 (30.8)	179 (31.2)	0.99 (0.77 to 1.27)	0.916
Multiple pregnancy rate per positive pregnancy (including twins)	11 (15.3)	34 (15.5)	0.98 (0.53 to 1.84)	0.960
Preterm delivery among clinical pregnancies	9 (13.4)	27 (12.5)	1.08 (0.53 to 2.17)	0.841
Singleton birthweight (g), mean ± SD	3268.1 ± 484.1	3291.8 ± 478.8	–	0.720
Small for gestational age *	3 (5.4)	11 (6.1)	0.87 (0.25 to 3.02)	>0.999
Large for gestational age *	6 (10.7)	16 (8.9)	1.20 (0.49 to 2.92)	0.691
Gestational diabetes among clinical pregnancies	3 (4.5)	15 (6.4)	0.65 (0.19 to 2.16)	0.663
Gestational hypertension among clinical pregnancies	5 (7.5)	19 (8.8)	0.85 (0.33 to 2.19)	0.732

**Abbreviation:** LPS, luteal phase support; CI, confidence interval.

*Small and large for gestational age defined as less than or more than two standard deviation units from expected birth weight. Small and large for gestational age were calculated from growth curves for Scandinavian children adjusted for sex and gestational age.

Data are presented as numbers (%).

### 3.4 Multivariate regression analysis of LBR

According to Table 4, binary logistic regression analysis (allowing for confounder correction) revealed no significant connection between timing of LPS initiation after hCG triggering in mNC-FET cycles and LBR in the adjusted model (adjusted OR 0.98, 95% CI 0.67–1.43, P = 0.913). Female age at oocyte retrieval (≥35 yrs. vs. < 35 yrs, adjusted OR 0.62, 95% CI 0.44–0.89, P = 0.009), BMI (≥25 kg/m^2^ vs. < 25 kg/m^2^, adjusted OR 0.63, 95% CI 0.42–0.94, P = 0.024), embryo transfer stage (D5/D6 vs. D3, adjusted OR 5.32, 95% CI 2.05–13.81, P < 0.001), physicians of embryo transfer (physician D vs. physician A, adjusted OR 0.43, 95% CI 0.27–0.69, P < 0.001), and number of embryos transferred (Double vs. Single, adjusted OR 5.12, 95% CI 2.13–12.33, P < 0.001) were all independent factors in LBR.

**Table 4:** Crude and adjusted OR for the timing of LPS initiation in mNC-FETs and other key potential confounders for LBRs.

Variable	Crude OR (95% CI) *	Adjusted OR (95% CI)	Adjusted P-value
Timing of LPS initiation in mNC-FETs			
Premature LPS group	Reference	Reference	
Conventional LPS group	0.98 (0.68 to 1.41)	0.98 (0.67 to 1.43)	0.913
**Female age at oocyte retrieval**			
<35 yrs.	Reference	Reference	
≥35 yrs.	0.60 (0.43 to 0.84)	0.62 (0.44 to 0.89)	**0.009**
**Body mass index (BMI)**			
<25 kg/m^2^	Reference	Reference	
≥25 kg/m^2^	0.65 (0.44 to 0.95)	0.63 (0.42 to 0.94)	**0.024**
**FSH on menstrual cycle days 2**–**3**			
≤10 mIU/ml	Reference	Reference	
>10 mIU/ml	0.90 (0.54 to 1.50)	1.06 (0.61 to 1.86)	0.829
**LH on menstrual cycle days 2**–**3**			
≤7 mIU/ml	Reference	Reference	
>7 mIU/ml	0.63 (0.40 to 1.00)	0.65 (0.40 to 1.06)	0.082
**Cycle outcomes**			
Freeze-all cycles	Reference	Reference	
Fresh ETs	1.12 (0.77 to 1.61)	1.34 (0.90 to 2.01)	0.148
**Number of previous ET cycles**			
<3	Reference	Reference	
≥3	0.75 (0.50 to 1.14)	0.71 (0.45 to 1.11)	0.129
**LH level on LH surge day**			
<15 mIU/ml	Reference	Reference	
15–24.9 mIU/ml	0.78 (0.51 to 1.21)	0.79 (0.50 to 1.25)	0.316
25–39.9 mIU/ml	0.96 (0.62 to 1.48)	1.01 (0.64 to 1.60)	0.955
≥40 mIU/ml	1.27 (0.84 to 1.94)	1.31 (0.85 to 2.04)	0.225
**P**_**4**_**level on LH surge day**			
<1.0 ng/ml	Reference	Reference	
≥1.0 ng/ml	0.99 (0.71 to 1.38)	0.95 (0.67 to 1.35)	0.774
**Luteal phase support**			
Non-mixed	Reference	Reference	
Mixed	1.18 (0.84 to 1.66)	1.21 (0.84 to 1.73)	0.303
**P**_**4**_**level on ET day**			
<10 ng/ml	Reference	Reference	
≥10 ng/ml	1.27 (0.74 to 2.17)	1.15 (0.65 to 2.02)	0.633
**Assisted hatching**			
Yes	Reference	Reference	
No	1.11 (0.81 to 1.51)	1.15 (0.82 to 1.61)	0.425
**Embryo transfer stage**			
D3	Reference	Reference	
D5/D6	1.04 (0.68 to 1.57)	5.32 (2.05 to 13.81)	**< 0.001**
**Number of embryos transferred**			
Single	Reference	Reference	
Double	1.61 (1.11 to 1.35)	5.12 (2.13 to 12.33)	**< 0.001**
**Good-quality embryo transferring**			
Yes	Reference	Reference	
No	0.74 (0.54 to 1.00)	0.74 (0.53 to 1.05)	0.094
**Physicians of embryo transfer**			
Physician A	Reference	Reference	
Physician B	0.82 (0.54 to 1.24)	0.77 (0.50 to 1.19)	0.241
Physician C	0.91 (0.60 to 1.39)	0.88 (0.57 to 1.37)	0.568
Physician D	0.42 (0.29 to 0.73)	0.43 (0.27 to 0.69)	**< 0.001**
**Endometrial thickness prior to FET**			
<8 mm	Reference	Reference	
≥8 mm	1.14 (0.69 to 1.65)	1.13 (0.67 to 1.93)	0.640

**Abbreviation:** LPS, luteal phase support; OR, odds ratio; CI, confidence interval; mNC, modified natural cycle; FET, frozen embryo transfer; LBR, live birth rate; FSH, follicle stimulating hormone; LH, luteinizing hormone.

* No adjustments for other covariates.

Statistically significant values are indicated in bold.

### 3.5 Sensitivity analysis

No significant statistical differences were found between the premature and conventional LPS groups of subsequent sensitivity analyses conducted for LBR according to the serum LH and P_4_ levels on LH surge day (P > 0.05 for all, see Table 5).

**Table 5:** Sensitivity analysis according to the serum LH and P_4_ levels on LH surge day.

	Premature LPS		Conventional LPS		Odds ratio (95% CI)	P-value
	Total No.	Events (%)	Total No.	Events (%)		
**Total**	182	56 (30.8)	574	179 (31.2)	0.99 (0.77 to 1.27)	0.916
**LH level on LH surge day**						
<15 mIU/ml	88	27 (30.7)	264	83 (31.4)	0.97 (0.57 to 1.63)	0.894
15–24.9 mIU/ml	30	10 (33.3)	111	27 (24.3)	1.56 (0.65 to 3.73)	0.320
25–39.9 mIU/ml	35	8 (22.9)	97	32 (33.0)	0.60 (0.25 to 1.47)	0.264
≥40 mIU/ml	29	11 (37.9)	102	37 (36.3)	1.07 (0.46 to 2.52)	0.870
**P**_**4**_**level on LH surge day**						
<1.0 ng/ml	131	44 (33.6)	392	119 (30.4)	1.16 (0.76 to 1.77)	0.489
≥1.0 ng/ml	51	12 (23.5)	182	60 (33.0)	0.63 (0.31 to 1.28)	0.197

**Abbreviation:** LPS, luteal phase support; P_4_, progesterone; LH, luteinizing hormone; CI, confidence interval.

## 4 Discussion

We conducted this study to determine if the LBRs in mNC-FET are negatively impacted by the premature timing of LPS initiated by P4 24 h after hCG triggering. As a result, the data imply that the premature timing of P4 LPS initiation has no effect on the LBR in infertile patients using modified natural cycle to prepare endometrium for FET. In mNC-FE, premature LPS initiation had no negative impacts on the cycle outcomes. Thus, we draw the conclusion that patients undergoing mNC-FET can have flexible scheduling for embryo transfer procedure as long as sufficient time was allowed for endometrial exposure to exogenous progesterone.

Recent studies suggested that mNC-FET is preferable to HRT-FET for patients with regular ovulation in terms of pregnancy outcomes [27]. In contrast to HRT-FET, mNC-FET has a lower miscarriage rate, according to several previous studies [28,29,30,31]. Additionally, mNC-FET is associated with a lower risk of pregnancy complications such as prenatal hypertension and intrahepatic cholestasis, as well as a lower risk of large-for-gestational-age babies in singletons and more favorable postnatal outcomes when compared to HRT-FET [32,33]. Nevertheless, mNC-FET has fewer scheduling options for embryo transfer dates than HRT-FET, which limits its clinical use. Therefore, we performed this retrospective analysis to explore the timing of progesterone LPS for mNC-FET in order to provide doctors and IVF labs greater scheduling flexibility in order to optimize patient benefit.

In this study, all patients chose to revive D3 embryos or blastocysts for transfer on day 4 or 6 after P4 administration. Synchronous development of the embryo and receptive endometrium is essential for successful implantation [15]. Previous studies have shown that embryo implantation rates are significantly reduced when asynchrony of the embryo and endometrium is greater than ±1.5 days [34]. In 2021, Weiss et al. conducted a proof-of-concept trial in which 56 patients treated with NC-FET underwent D2 cleavage-stage embryo transfer two days following P4 injection, regardless of ovulation or LH surge, and achieved a promising pregnancy outcome [35]. This shows that superior pregnancy outcomes can be obtained even in the absence of ovulation, provided that synchronization between the embryo and endometrium is maintained. Moreover, a recent meta-analysis suggested that day 3 embryos and blastocysts should be transferred after a full 3 and 5 days of P4 treatment in order to achieve higher implantation rates and improve embryo and endometrial synchrony [15]. There may be an explanation for the previous finding of Kyrou et al. that adding LPS 24 h after the hCG trigger did not increase ongoing pregnancy rates (22% vs. 21%, p = 0.8) among FET patients compared with those without LPS treatment [21]. This may be due to the fact that the aforementioned study fixed the time of embryo transfer as 5 days after the hCG trigger, while the LPS group initiated LPS 24 h after the hCG trigger, which could result in asynchrony between the endometrium and the embryo.

At the moment, the duration of LPS in clinical practice is ambiguous, and further research is needed to investigate this issue. Some clinics elected to discontinue LPS on the day of a positive pregnancy test, whereas 52% preferred to maintain LPS until 12 weeks' gestation [36]. Despite a paucity of relevant evidence, it appears that there is a preference for continuing P4 supplementation until 8–10 weeks of gestation [37,38]. In our center, if a clinical pregnancy is verified, LPS will be continued until the placenta completely replaces luteal function at 8–10 weeks of gestation.

A debate exists regarding whether LH levels on the day of hCG triggering affect clinical outcomes, and there is no consensus as to the appropriate level of LH for hCG triggering during mNC-FET cycles [39]. In a retrospective study by Reichman et al. it was discovered that when hCG was administered within one day of the LH surge (LH > 17 IU/L, E2 level dropped), patients undergoing mNC-FET cycles achieved a superior ongoing pregnancy rate [40]. Another study involved 233 patients who underwent mNC-FET using D4 embryos. In all patients, hCG was triggered once the dominant follicle was greater than 17 mm in diameter and the endometrium was acceptable. Five days after hCG triggering, FET was performed. In their study, the researchers found that spontaneous LH surge (LH > 10 IU/L) did not impact pregnancy outcomes [41]. Subsequently, A retrospective study conducted by Kahraman and his colleagues in 2020 found that hCG can be administered between the start of the LH rise (15 IU/L) and the LH peak (40 IU/L) without negatively impacting the clinical outcome of mNC-FET [42]. However, in a study of modified natural cycle for frozen-thawed euploid blastocyst transfer, an increase in LH ≥ 13 mIU/ml before the administration of hCG was found to have a negative impact on clinical pregnancy rates, and the researchers advised that in the presence of LH level ≥13 mIU/ml, it is recommended to avoid hCG administration and to plan the embryo transfer only after spontaneous follicular ruptures have occurred [43]. A possible explanation for this is the extremely small sample size of the LH ≥ 13 group (n = 22). Consequently, we performed an additional sensitivity analysis to assess the potential impact of LH and P4 levels on LBR on hCG trigger day and to confirm the reliability of the results, and found no significant statistical difference between groups.

For mNC-FET, there is now debate over when to add P4 following hCG triggering, and no research has explored its influence on LBR. Our study fills this need and is novel. In spite of this, there are some limitations to this study. Firstly, due to the fact that it is a retrospective analysis conducted by a single center, this study is biased. Furthermore, we do not anticipate monitoring patients for follicular rupture or ovulation after hCG triggering. Further research is required to corroborate our findings in the future. Despite these limitations, our study findings are still useful for doctors using customizable mNC-FET methods.

## 5 Conclusion

In summary, our study demonstrated that adding exogenous P4 for LPS 24 h after hCG triggering does not adversely affect mNC-FET cycle outcomes provided enough time is allowed for endometrium exposure to exogenous P4. This provides clinicians with a more flexible choice regarding embryo transfer date scheduling. However, more prospective clinical trials need to be designed to further validate our findings.

## Author contribution statement

Jing-Yan Song: Conceived and designed the experiments; Performed the experiments; Analyzed and interpreted the data.

Zhen-Gao Sun: Conceived and designed the experiments.

Wen-Jing Jiang: Performed the experiments; Analyzed and interpreted the data; Contributed reagents, materials, analysis tools or data; Wrote the paper.

## Funding statement

This research did not receive any specific grant from funding agencies in the public, commercial, or not-for-profit sectors.

## Data availability statement

Data will be made available on request

## Declaration of interest’s statement

The authors declare no conflict of interest.
